# Antinociceptive and Anti-Inflammatory Effects of Ethanolic Extracts of *Glycine max* (L.) Merr and *Rhynchosia nulubilis* Seeds

**DOI:** 10.3390/ijms10114742

**Published:** 2009-11-04

**Authors:** Joo Hyuk Yim, Ok-Hwan Lee, Ung-Kyu Choi, Young-Chan Kim

**Affiliations:** 1 Korea Food Research Institute, Seongnam, Kyonggi, 463-746, Korea; E-Mail: jlunar@naver.com (J.H.Y.); 2 Department of Biomedical Science, CHA University, Seongnam, Kyonggi, 463-836, Korea; 3 Pohang Center for Evaluation of Biomaterials, Pohang 790-834, Korea; E-Mail: cuk8272@hanmail.net (U.-K.C.)

**Keywords:** Glycine max, Rhynchosia nulubilis, anti-inflammatory activity, anti-nociceptive activity

## Abstract

The aim of this study was to assess the *in vivo* potential of ethanolic extracts of *Glycine max* (L.) Merr. (SoRiTae) and *Rhynchosia nulubilis* (Yak-Kong) seeds as natural anti-nociceptive and anti-inflammatory agents. To assess the anti-nociceptive and anti-inflammatory potential, the ethanolic extracts of SoRiTae and Yak-Kong seeds were tested in arachidonic acid-induced ear edema, carrageenan induced paw edema, formalin-induced licking time, acetic acid induced writhing and hot plate-induced thermal stimulation in mice. The administration of ethanolic extracts of SoRiTae and Yak-Kong seeds evoked a significant effect of anti-nociceptive and anti-inflammatory activities as compared to standards aminopyrine and indomethacin. The ear edema, paw edema, paw licking time, pain and writhes in mice were significantly reduced (*p* < 0.05) as compared to the control. The results obtained in this study indicate that both SoRiTae and Yak-Kong soybeans possesses potential anti-nociceptive and anti-inflammatory activities.

## 1. Introduction

Black soybeans have been widely used as materials for traditional oriental medicines, unlike yellow soybeans, which have been mostly used as food materials. Medicinal herbs have been used as a form of therapy for the relief of pain throughout history [1]. The treatment of rheumatic disorders is an area in which the practitioners of traditional medicine enjoy patronage and success [2]. Natural products in general and medicinal plants in particular, are believed to be an important source of new chemical substances with potential therapeutic efficacy. Taking into account that the most important anti-nociceptive and anti-inflammatory drug prototypes were originally derived from the plant sources, the study of plant species traditionally used as pain killers should still be seen as a fruitful research strategy in the search of new anti-nociceptive and anti-inflammatory drugs.

Black soybeans, *Glycine max* (L.) Merr. also known as SoRiTae and *Rhynchosia nulubilis* (Yak-Kong) are species of legume native to East Asia. These soybeans are cultivars with a black seed coat. In traditional Chinese medicine, black soybeans have been used for detoxification, as an anti-inflammatory, and to improve the fluidity of blood [3]. They contain flavonoid and non-flavonoid molecules, including anthocyanins, and owing to the rich anthocyanin contents in their seed coat they are potentially important sources for the development of natural antioxidants [4]. Anthocyanins, like other polyphenolic substances, can effectively scavenge oxygen free radicals. SoRiTae is reported to have multiple therapeutic properties like anti-tumor and antioxidant activities for improving the fluidity of the whole blood [5]. A control case study reported that black soybean consumption reduced the risk of breast cancer in Korean women [6]. Daily intakes of black soybean were also associated with a reduced cardiovascular disease risk [7]. Furthermore, an aqueous infusion of black soybean has been traditionally used in folk medicine for treating pharyngolaryngeal symptoms in acute respiratory diseases such as sore throats, and for improving hoarseness and sputum expectoration [8].

However, there is no scientific report available in the literature on the anti-nociceptive and anti-inflammatory activities of the ethanolic seed extracts of SoRiTae and Yak-Kong. In view the fact that plants are used by traditional healers to alleviate pain in patients, the present study therefore aimed to determine the anti-nociceptive and anti-inflammatory activities of the ethanolic seed extracts of SoRiTae and Yak-Kong, in relation with their folklore medicinal properties.

## 2. Results and Discussion

### 2.1. Arachidonic acid-induced ear edema

**Figure 1.**
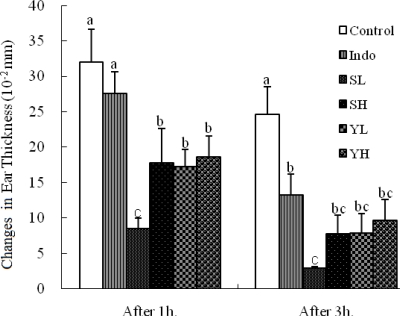
Effects of SoRiTae and Yak-Kong ethanolic seed extracts on arachodonic acid-induced ear edema. Indo: Indomethacin (10 mg/kg); SL: SoRiTae (100 mg/kg); SH: SoRiTae (200 mg/kg), YL: Yak-Kong (100 mg/kg), YH: Yak-Kong (200 mg/kg). Each values was expressed as the mean ±S.E.M. (n = 6). ^a–c^ Means in the same column not sharing a common letter are significantly different (p < 0.05) by Duncan's multiple test.

The anti-inflammatory activities of ethanolic extracts of SoRiTae and Yak-Kong were measured on mouse ear by using arachidonic acid as an inducer. It was found that ethanolic seed extracts of SoRiTae and Yak-Kong caused significant inhibition (*p* < 0.05) of edema as compared to the control group (Figure 1). Furthermore, the inhibitory potency of the ethanolic seed extracts of SoRiTae at low concentration (100 mg/kg) was better than that of indomethacin (10 mg/kg) at 3 h after induction.

### 2.2. Carrageenan-induced paw edema

**Figure 2.**
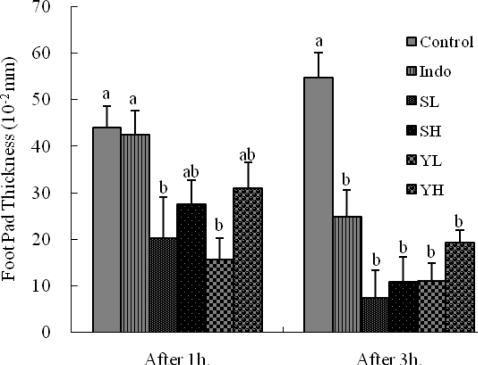
Effects of SoRiTae and Yak-Kong ethanolic seed extracts on carrageenan-induced paw edema. Indo: Indomethacin (10 mg/kg); SL: SoRiTae (100 mg/kg); SH: SoRiTae (200 mg/kg), YL: Yak-Kong (100 mg/kg), YH: Yak-Kong (200 mg/kg). Each values was expressed as the mean ±S.E.M. (n = 6). ^a–b^ Means in the same column not sharing a common letter are significantly different (p < 0.05) by Duncan's multiple test.

The results of the anti-inflammatory effect of the ethanolic extracts of SoRiTae and Yak-Kong on carrageenan-induced paw edema in mice right hind paws are presented in Figure 2. There was a gradual increase in edema paw volume of mice in the control group (carrageenan treated). However, in the test groups treated with ethanolic seed extracts of SoRiTae and Yak-Kong, showed a significant reduction in the edema paw volume. The ethanolic seed extracts of SoRiTae and Yak-Kong at the concentration of 200 mg/kg exhibited lesser inhibitory effect, 1 h after injecting indomethacin as compared to the ethanolic extracts at 100 mg/kg. However, there was no significant pattern of edema paw volume was observed, 3 h after injection of indomethacin using 100 and 200 mg/kg of SoRiTae and Yak-Kong ethanolic seed extracts.

### 2.3. Formalin-induced paw licking

**Figure 3.**
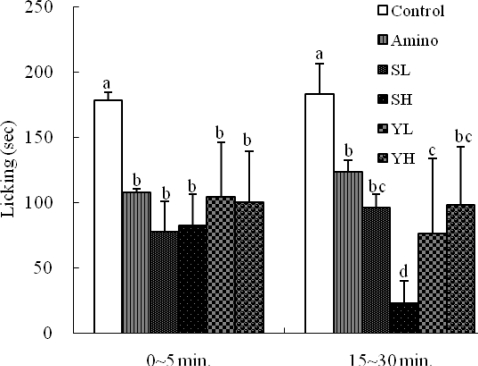
Effects of SoRiTae and Yak-Kong ethanolic seed extracts on formalin-induced paw licking in mice. Amino: Aminopyrine (50 mg/kg); SL: SoRiTae (100 mg/kg); SH: SoRiTae (200 mg/kg), YL: Yak-Kong (100 mg/kg), YH: Yak-Kong (200 mg/kg). Each values was expressed as the mean ±S.E.M. (n = 6). ^a–d^ Means in the same column not sharing a common letter are significantly different (p < 0.05) by Duncan's multiple test.

The anti-nociceptive profile of ethanolic seed extracts of SoRiTae and Yak-Kong, measured on mouse paw by using injection of formalin solution is shown in Figure 3. The extracts exhibited significant dose related reduction (*p* < 0.05) of hind paw licking caused by formalin. Interestingly, the ethanolic seed extract of SoRiTae at the concentration of 200 mg/kg exhibited complete inhibitory effect in late phase.

### 2.4. Abdominal constriction response caused by acetic acid

**Figure 4.**
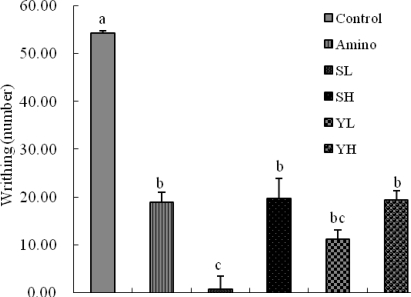
Effects of SoRiTae and Yak-Kong ethanolic seed extracts on acetic acid-induced writhing in mice. Amino: Aminopyrine (50 mg/kg); SL: SoRiTae (100 mg/kg); SH: SoRiTae (200 mg/kg), YL: Yak-Kong (100 mg/kg), YH: Yak-Kong (200 mg/kg). Each values was expressed as the mean ±S.E.M. (n = 6). ^a–c^ Means in the same column not sharing a common letter are significantly different (p < 0.05) by Duncan's multiple test.

Acetic acid induced writhing test was used for detecting both central and peripheral analgesia. The effects of different doses of the ethanolic seed extracts of SoRiTae and Yak-Kong on the number of writhes/stretches movement induced by acetic acid in mice are shown in Figure 4. Administration of extracts (100–200 mg/kg) significantly (*p* < 0.05) reduced the number of writhes induced by the injection of acetic acid in mice as compared to control group. SoRiTae (100 mg/kg) showed better results of inhibitory effect as compared to control, which were also comparable with the reference drug aminopyrine (positive control).

### 2.5. Thermally-induced pain in mice

**Figure 5.**
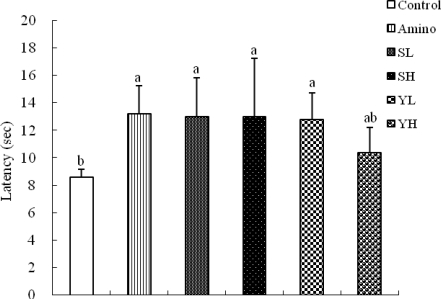
Effects of SoRiTae and Yak-Kong ethanolic seed extracts on thermally-induced pain in mice. Amino: Aminopyrine (50 mg/kg); SL: SoRiTae (100 mg/kg); SH: SoRiTae (200 mg/kg), YL: Yak-Kong (100 mg/kg), YH: Yak-Kong (200 mg/kg). Each values was expressed as the mean ±S.E.M. (n = 6). ^a–b^ Means in the same column not sharing a common letter are significantly different (p < 0.05) by Duncan's multiple test.

Animal groups treated with the ethanolic seed extracts of SoRiTae and Yak-Kong (100–200 mg/kg) elicited an increase in the latency response in the hot plate test (Figure 5). These increases in latency responses (analgesic effect) were statistically significant (*p* < 0.05). The results showed that SoRiTae and Yak-Kong ethanolic seed extracts (100 and 200 mg/kg) produced a significant anti-nociceptive action when compared to the control group.

### 2.6. Discussion

According to our findings, the ethanolic extracts of SoRiTae and Yak-Kong produced potential antinociceptive and anti-inflammatory effects when assessed in both chemical and thermal methods (to detect central and peripheral analgesics) of nociception including arachidonic acid-induced ear edema, carrageenan induced paw edema, formalin-induced paw edema, acetic acid induced writhing and hot plate-induced thermal stimulation in experimental ICR mice.

As in the arachidonic acid-induced ear edema and acetic acid-induced writhing, a dose related antinociceptive effect of the extracts was observed. Arachidonic acid-induced ear edema is a reliable method used to evaluate lipoxygenase inhibitors [9]. In arachidonic acid-induced ear edema, the ethanolic extracts of SoRiTae and Yak-Kong significantly (*p* < 0.05) inhibited the ear edema in tested experimental mice as compared to control group (Figure 1). These results suggest that the plant extracts attenuate the pain by inhibition of cycloxygenase and lipoxygenase in the arachidonic acid pathway. Moreover, these results are in good agreement with another study by Reanmongkol *et al*. [10], in which the extract of from plant source as a *Putranjiva roxburghii* inhibited croton oil-induced ear edema in a dose-dependent manner (1.25, 2.5, and 5.0 mg/ear) in mice, and decreased anus edema induced by croton oil at the high dose of 800 mg/kg in rats.

Acetic acid induces inflammatory pain by inducing capillary permeability [11], formalin exhibits neurogenic and inflammatory pain [12], while hot plate-induced pain indicates narcotic involvement [13]. Collier *et al.* [14] proposed that the acetic acid acts indirectly by inducing the release of endogenous mediators which stimulate the nociceptive neurons sensitive to non-steroidal anti-inflammatory drugs (NSAIDs) and opioids. Both ethanolic extracts (100 and 200 mg/kg) administered orally on inflamed or non inflamed paws, significantly reduced the increase in carrageenan-induced paw edema (Figure 2). The ethanolic extracts of SoRiTae and Yak-Kong showed significant anti-inflammatory activity at all dose levels studied, as compared with control group. Since the topical application of the extracts on the non-oedematous paw reduced the inflammation of the oedematous paw, it may be assumed that the anti-inflammatory principle(s) might have been absorbed transdermally to produce a systemic effect. Development of edema induced by carrageenan is commonly correlated with the early exudates-stage of inflammation, one of the important processes of inflammatory pathology [15]. As shown in Figure 2, after 1 h the inflammation increased gradually and was elevated for the later 3 h. This could be attributed to the liberation of prostaglandins and kinins, which accompany leukocyte migration [16]. In the formalin test, a distinctive nociceptive response is termed in early and late phases. The anti-nociceptive action in the formalin test of both extracts paralleled with inhibitory effect of acetic acid-induced writhing. This model produces a distinct biphasic nociception.

Drugs that act primarily on the central nervous system inhibit both phases equally while peripherally acting drugs inhibit the late phase [17,18]. The early phase is probably a direct result of stimulation of nociceptors in the paw which reflects centrally mediated pain while the late phase is due to inflammation with a release of serotonin, histamine, bradykinin and prostaglandins [19]. As shown in Figure 3, the suppression of both phases of pain, as observed in this study by administration of ethanolic extracts of SoRiTae and Yak-Kong also reflected strong possibilities of having both central and peripheral effects. In addition, the extract of SoRiTae (200 mg/kg) significantly reduced compared to other samples in late phase. Both carrageenan-induced edema and formalin induced late phase inflammations correlated with a release of histamine and bradykinin. In case of the ethanolic extracts of SoRiTae and Yak-Kong, SoRiTae extract exhibited better activity in late phase than early phase. These phases have different properties and are very useful tools, not only for assessing the potency of analgesic, but also for elucidating the mechanisms of pain and analgesia. However, the action of analgesic is different in the early (neurogenic) and late (inflammatory) phase. Acetic acid induced writhing test was used for detecting both central and peripheral analgesia. Intraperitoneal administration of acetic acid releases prostaglandins and sympathomimetic system mediators like PGE_2_ and PGF_2α_ with increased levels of peritoneal fluid in acetic acid induced mice [20].

As shown in Figure 4, the abdominal constrictions produced after administration of acetic acid might be related to sensitization of nociceptive receptors to prostaglandins. It is, therefore possible that the ethanolic extracts of SoRiTae and Yak-Kong exerted their analgesic effect probably by inhibiting the synthesis or action of prostaglandins. Hot plate test is a specific central antinociceptive test. The ethanolic extracts of SoRiTae and Yak-Kong exhibited significant anti-nociceptive activity in hot plate method as compared to control group (Figure 5). This effect might be inhibited by naloxone. Thus, these results indicate that both extracts may exert their effects through central opioid receptors or promoted release of endogenous opiopeptides. Thermal nociceptive tests are more sensitive to opioid μ receptors and non-thermal to opioid κ receptors [21].

In the present study, the ethanolic extracts of SoRiTae and Yak-Kong significantly exhibited anti-inflammatory effects. As inflammation is a peripheral process, therefore, t is suggested that the extract also exerted peripheral effects. In fact, the licking activity in the formalin test was strongly diminished in the second phase, whereas this reduction was more discreet in the first phase. In this view, the extract seems to act by both central and peripheral mechanisms. Further, recent research suggest that the black soybean seeds revealed the presence of phytochemical such as anthocyanins, proanthocyanidins, and flavonoids [22], which have potential antinociceptive and anti-inflammatory effects. In addition, several studies demonstrated that bioactive flavonoids such as rutin, quercetin, luteolin, hesperidin, as well as biflavonoids produce significant antinociceptive and/or anti-inflammatory activities [23,24]. Thus, further studies will be necessary to understand the mechanisms of action underlying the effects of the extract and their active compounds.

## 3. Experimental Section

### 3.1. Plant material and preparation of ethanolic extracts

The seeds of SoRiTae and Yak-Kong were purchased from a local Nonghyup market in Seoul, South Korea. The seeds of SoRiTae and Yak-Kong were pulverized into a dry powder and extracted separately with 70% ethanol (Mallinckordt Baker, Phillipsburg, NJ, USA). The crude extracts were filtered, evaporated under rotary evaporator (Sunileyela, Kyonggi, S. Korea) at room temperature (below 50 ºC), and then freeze dried (Ilshin, Kyonggi, S. Korea) to give the mass with percentage yields of 11.2 and 12.83% (w/w), respectively.

### 3.2. Experimental animals

Animal use protocol was approved by Kyoung-Hee University, South Korea (*Animal Eths Comm/IE/98/Reg No 379/01/ab/CPCSEA*) and was in accordance with International Standard on the care and use of experimental animals [25]. Male ICR mice (six weeks old) were purchased from Central Lab. Animal Inc. (Seoul, South Korea). Animals were maintained under constant temperature (24 ± 2 ºC), 12 h light-dark cycle, relative humidity 40–70%, fed with food (Purina, Seoul, South Korea) and water ad *libitum* and fasted overnight (18 h) before the day of the experiment.

### 3.3. Test samples/drugs

Accurately weighed quantities of the ethanolic seed extracts of SoRiTae and Yak-Kong were suspended in 0.5% sodium carboxymethyl cellulose (CMC-Na) solution (Sigma, St. Louis, MO, USA) to prepare suitable forms of the dosages, immediately before the start of experiments. Mice were randomly divided into six groups (n = 6) and administered orally with the ethanolic seed extracts (100 and 200 mg/kg) of SoRiTae and Yak-Kong. Indomethacin (10 mg/kg) and aminopyrine (50 mg/kg) (Yooyoung Pharm., Seoul, S. Korea) were used as positive controls while 0.5% CMC-Na was served as a negative control.

### 3.4. Arachidonic acid-induced ear edema

The method of Kim *et al*. [26] was adopted for this assay. The ethanolic seed extracts (100 and 200 mg/kg) of SoRiTae and Yak-Kong were orally administered at 1 h prior to the topical application of 2% arachidonic acid dissolved in acetone to right ear of mice (0.02 mL/ear), then ear thickness was measured using a Dial Thickness Gauge (Mitutoyo, Tokyo, Japan) before and after at 1 and 3 h arachidonic acid treatment, and then differences in the thickness were calculated. The degree of ear swelling was expressed as an increase in ear thickness (mm).

### 3.5. Carrageenan-induced paw edema

The procedure used to assess anti-inflammatory activity was based on the method used by Winter *et al.* [27] At 1 h after sample (SoRiTae and Yak-Kong ethanolic seed extracts) administration, edema was induced by injecting 0.02 mL of 1% carrageenan in sterile saline into the plantar side of the right hind paw. The pad thickness of hind paw was measured with a Dial Thickness Gauge before and at 1 and 3 h after carrageenan injection and the differences in the thickness were calculated. The degree of foot-pad swelling was expressed as an increase in foot-pad thickness (mm).

### 3.6. Formalin-induced paw licking in mice

The procedure was essentially similar to previously described method of Correa and Calixto [28]. Twenty microgram of 2.5% formalin solution (Sigma) was injected subcutaneously under the surface of the right hind paw for the induction of pain. The amount of time spent in licking the injected paw was monitored, and was considered as an indicative of pain. The first of the nociceptive response normally peaked 5 min after formalin injection and the second phase 15–30 min after formalin injection, representing the neurogenic and inflammatory pain responses, respectively. The animals were pretreated with samples (SoRiTae and Yak-Kong ethanolic seed extracts) at 1 h, before being challenged and buffered formalin, and the responses were observed for 30 min.

### 3.7. Acetic acid-induced writhing in mice

Method of Besra *et al.* [29] was adopted for this study. The abdominal constrictions resulting from intraperitoneal (i.p.) injection of acetic acid (3%) consisting of the contraction of abdominal muscle together with a stretching of hind limbs. The animals were pretreated with samples (SoRiTae and Yak-Kong ethanolic seed extracts), and then after 1 h, acetic acid was administered (i.p.). Antinociceptive effects of the ethanolic seed extracts from SoRiTae and Yak-Kong were recorded by counting the total number of writhes. The number of writhes was counted over a period of 5 min after acetic acid injection. The data represented the total number of writhes observed during 10 min and was expressed as writhing numbers. A writhe is indicated by abdominal constriction and full extension of hind limb.

### 3.8. Thermally-induced pain in mice

The hot plate test was used to measure the response latencies based on the method of Vaz *et al.* [30]. In this experiment, hot plate (Corning, NY, USA) was maintained at a temperature of 60 ± 1 °C. The basal reaction time of all animals towards thermal heat was recorded. The animals which showed for paw licking or jumping response within 6–8 seconds were selected for the study. The animals in all the groups were individually exposed to the hot plate maintained at 60 °C, 1 h after the administration of test samples and reference compounds. The time taken in seconds for fore paw licking or jumping was taken as the index of response latency. An automatic 30 seconds cut-off was used to prevent tissue damage.

### 3.9. Statistical analysis

The data were expressed as mean ±SEM of 6 animals. Statistical analysis was carried out using one-way ANOVA followed by Duncan's multiple range test. All statistical analyses were carried out by using SAS statistical software. The differences were considered significant at *p* < 0.05.

## 4. Conclusions

Based on the above results, we have confirmed that the ethanolic seed extracts of SoRiTae and Yak-Kong have variable degrees of antinociceptive properties. The extracts also have activity against inflammation. Our results suggest that SoRiTae and Yak-Kong might be an excellent natural food ingredient, and may also useful for the development of antinociceptive and anti-inflammatory agents.
